# Supplementary material for: Complexome profiling of the Chlamydomonas psb28 mutant reveals TEF5 as an early PSII assembly factor
Source: Plant Cell. 2025 Mar 17;37(6):koaf055. doi: 10.1093/plcell/koaf055 (PMC12164590; doi:10.1093/plcell/koaf055)
Supplement: koaf055_Supplementary_Data [file koaf055_supplementary_data.zip › psb28_interactive.zip!/plots/intensity/qualityPlots/peptides/SAH1_Cre03.g204250.t1.2.html]

### Cre03.g204250.t1.2

Trivial name: SAH1  
SeqCoverage [median]: 31.70  
Mass: 52.72 kDa  
  
 

| Sequence | MissedCleavage | Mass [Da] | wtIntensity | muIntensity | Score |
| --- | --- | --- | --- | --- | --- |
| AEEQYAK | 0 | 837.39 | 987.48 | 1231.95 | 107.32 |
| ALSVDHVNGR | 0 | 1066.55 | 0.00 | 437.36 | 49.42 |
| ATDVMIAGK | 0 | 904.47 | 7757.39 | 10701.72 | 196.77 |
| DGTLPNPDSTDNAEFK | 0 | 1719.76 | 1505.08 | 4395.93 | 117.20 |
| DSAAVFAWK | 0 | 993.49 | 815.08 | 0.00 | 105.39 |
| FIFPDGHGVIVLAEGR | 0 | 1725.92 | 492.84 | 0.00 | 47.53 |
| IVLGLIR | 0 | 782.54 | 1395.09 | 0.00 | 71.07 |
| KVMGVSEETTTGVK | 1 | 1464.75 | 0.00 | 413.22 | 63.04 |
| KVMGVSEETTTGVKR | 2 | 1620.85 | 273.50 | 0.00 | 45.33 |
| LSADQAAYINVPVDGPYKPAHYR | 1 | 2544.28 | 0.00 | 2507.25 | 47.39 |
| QNIKPQVDR | 1 | 1096.60 | 0.00 | 620.57 | 53.76 |
| SEFGPAQPFK | 0 | 1106.54 | 0.00 | 1320.11 | 63.56 |
| SEFGPAQPFKGAK | 1 | 1362.69 | 0.00 | 1186.79 | 54.70 |
| VAALHLPK | 0 | 847.53 | 0.00 | 568.24 | 68.48 |
| VKDIAEADFGR | 1 | 1219.62 | 832.65 | 0.00 | 30.33 |
| VMGVSEETTTGVKR | 1 | 1492.76 | 0.00 | 2405.57 | 66.25 |
|  |  |  | 14059.1 | 25788.7 |  |
